# Trends in chemotherapy use for early-stage breast cancer from 2006 to 2019

**DOI:** 10.1186/s13058-024-01822-9

**Published:** 2024-06-13

**Authors:** Jenna Bhimani, Kelli O’Connell, Isaac J. Ergas, Marilyn Foley, Grace B. Gallagher, Jennifer J. Griggs, Narre Heon, Tatjana Kolevska, Yuriy Kotsurovskyy, Candyce H. Kroenke, Cecile. A. Laurent, Raymond Liu, Kanichi G. Nakata, Sonia Persaud, Donna R. Rivera, Janise M. Roh, Sara Tabatabai, Emily Valice, Erin J.A. Bowles, Elisa V. Bandera, Lawrence H. Kushi, Elizabeth D. Kantor

**Affiliations:** 1 Department of Epidemiology and Biostatistics, Memorial Sloan Kettering Cancer Center, 633 Third Avenue 3rd Floor, 10017 New York, NY USA; 2 Division of Research, Kaiser Permanente Northern California, Oakland, CA USA; 3 Department of Medicine, Division of Hematology/Oncology, Department of Health Management and Policy, University of Michigan, Ann Arbor, MI USA; 4 Department of Oncology, Kaiser Permanente Medical Center, Vallejo, CA USA; 5 San Francisco Medical Center, Kaiser Permanente Northern California, San Francisco, CA USA; 6 Kaiser Permanente Washington Health Research Institute, Kaiser Permanente Washington, Seattle, WA USA; 7 Division of Cancer Control and Population Sciences, National Cancer Institute, Rockville, MD USA; 8 Cancer Epidemiology and Health Outcomes, Rutgers Cancer Institute of New Jersey, New Brunswick, NJ USA

**Keywords:** Antineoplastics, Breast cancer, Chemotherapy, Epidemiology

## Abstract

**Background:**

Little is known about how use of chemotherapy has evolved in breast cancer patients. We therefore describe chemotherapy patterns for women with stage I-IIIA breast cancer in the Optimal Breast Cancer Chemotherapy Dosing (OBCD) Study using data from KPNC (Kaiser Permanente Northern California) and KPWA (Kaiser Permanente Washington).

**Findings:**

Among 33,670 women, aged 18 + y, diagnosed with primary stage I-IIIA breast cancer at KPNC and KPWA from 2006 to 2019, we explored patterns of intravenous chemotherapy use, defined here as receipt of intravenous cytotoxic drugs and/or anti-HER2 therapies. We evaluated trends in chemotherapy receipt, duration over which chemotherapy was received, and number of associated infusion visits. In secondary analyses, we stratified by receipt of anti-HER2 therapies (trastuzumab and/or pertuzumab), given their longer duration. 38.9% received chemotherapy intravenously, declining from 40.2% in 2006 to 35.6% in 2019 (p-trend < 0.001). Among 13,089 women receiving chemotherapy, neoadjuvant treatment increased (4.1–14.7%; p-trend < 0.001), as did receipt of anti-HER2 therapies (20.8–30.9%) (p-trend < 0.001). The average treatment duration increased (5.3 to 6.0 months; p-trend < 0.001), as did the number of infusion visits (10.8 to 12.5; p-trend < 0.001). For those receiving anti-HER2 therapies, treatment duration and average number of visits decreased; among those not receiving anti-HER2 therapies, number of visits increased, with no change in duration.

**Conclusions:**

While the prevalence of chemotherapy receipt has decreased over time, the use of neoadjuvant chemotherapy has increased, as has use of anti-HER2 therapies; duration and number of administration visits have also increased. Understanding these trends is useful to inform clinical and administrative planning.

## Introduction

Cytotoxic drugs were first recognized as treatment for early breast cancer in the 1970s [1]. Several groups of cytotoxic agents were identified as having overall survival benefit for early-stage breast cancer, including alkylating agents (cyclophosphamide), anti-metabolites (5-fluorouracil, methotrexate), anthracyclines (doxorubicin) and others [1]. These agents are recommended for prescribing by the NCCN (National Comprehensive Cancer Network) Breast Cancer Treatment Guidelines [2], along with anti-HER2 therapies for HER2+ (human epidermal growth factor 2)-positive disease.

For treatment of stage I-IIIA breast cancer (defined as early-stage breast cancer [EBC]), there are many treatment options; treatment can vary by drugs used, cycle length/interval and number of doses, manifesting in differing durations of chemotherapy and/or number of infusion visits. To this end, a given drug combination may be administered via different administration schedules (e.g., dose dense vs. standard schedules), some of which may be completed earlier, resulting in a shorter duration of treatment. Furthermore, certain drugs indicated for EBC have notably longer intended treatment duration than others, specifically anti-HER2 therapies, which are administered for up to a 1-year period [3, 4]. Thus, as guidelines change and regimens used in the population shift over time, and interest builds in the de-escalation of treatment, (5–6) it is unclear how all of this ultimately impacts the time in treatment and number of associated visits at a population-level. While previous studies have explored the impact of cytotoxic drugs on health service utilization, these studies have been focused on emergency department visits and unplanned admissions; [7–10] thus, research on infusion visits, which are often done in community settings, is lacking. This is important, as time in treatment and number of attendances at infusion centers represents a time and resource burden for patients and providers alike.

We therefore used data from Optimal Breast Cancer Chemotherapy Dosing (OBCD) study to examine trends in chemotherapy prescribing in a large cohort of women with EBC treated in community settings.

## Methods

### Study population

This analysis includes 33,670 women, aged 18+y, who were diagnosed with primary stage I-IIIA breast cancer at Kaiser Permanente Northern California (KPNC, 2006–2019) or Kaiser Permanente Washington (KPWA, 2006–2015). For this analysis, we defined ‘chemotherapy’ as receipt of cytotoxic chemotherapy and/or anti-HER2 therapy for the treatment of breast cancer. Participants were identified through the Kaiser Permanente Virtual Data Warehouse (VDW) [11], which includes data from each health system’s cancer registry. KPNC maintains its own cancer registry and reports data to the Greater Bay Area and Greater California Surveillance Epidemiology and End Results (SEER) Programs, while KPWA receives data from the Seattle/Puget Sound SEER Program.

Eligibility criteria included the following: diagnosed with primary breast cancer with no prior history or same day diagnosis of cancer (except non-melanoma skin cancer), enrolled at KPWA or KPNC at time of diagnosis, had available medical records, and did not opt their medical record out of research studies.

Information on patient demographics, clinical characteristics and chemotherapy was obtained from KPNC and KPWA. (11–12)

### Exposure and outcome

We examined four aspects of chemotherapy administration over time: known receipt of neoadjuvant treatment (yes vs no), receipt of neoadjuvant treatment (yes; no), duration over which chemotherapy was received (months), and chemotherapy administration visits (number of visits). We stratified analyses for chemotherapy receipt, chemotherapy duration and visits by receipt of anti-HER2 therapies, given their markedly longer duration of use in the guidelines. Linear and logistic regression models were used to evaluate temporal trends for continuous (duration of chemotherapy, number of infusion visits) and binary (receipt of chemotherapy, neoadjuvant chemotherapy and trastuzumab and/or pertuzumab) variables of interest, with linear trends in time measured with t-tests in the linear regression models and Wald tests in the logistic regression models. We also graphically show the distribution of neoadjuvant and adjuvant chemotherapy by hormone receptor status and HER2 status over time.

All analyses were conducted in SAS v9.4. IRB approval was obtained from all sites involved in the study (Memorial Sloan Kettering Cancer Center, KPNC, KPWA, and Rutgers), with a waiver of consent to collect patient data at KPNC and KPWA.

## Results

**Fig. 1:**
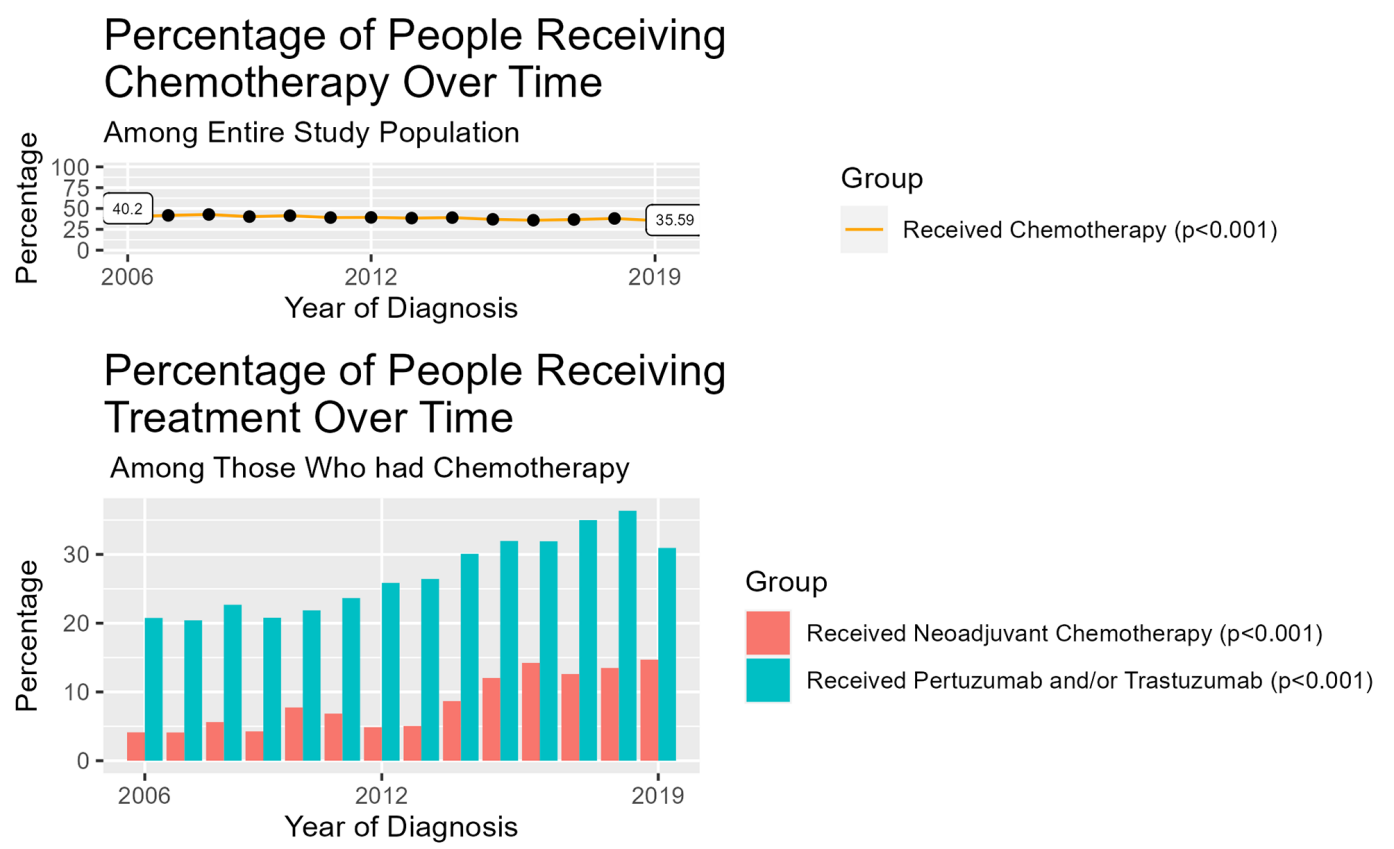
Over time, use of chemotherapy has decreased from 2006–2019 while use of neoadjuvant chemotherapy and trastuzumab/pertuzumab has increased

**Fig. 2:**
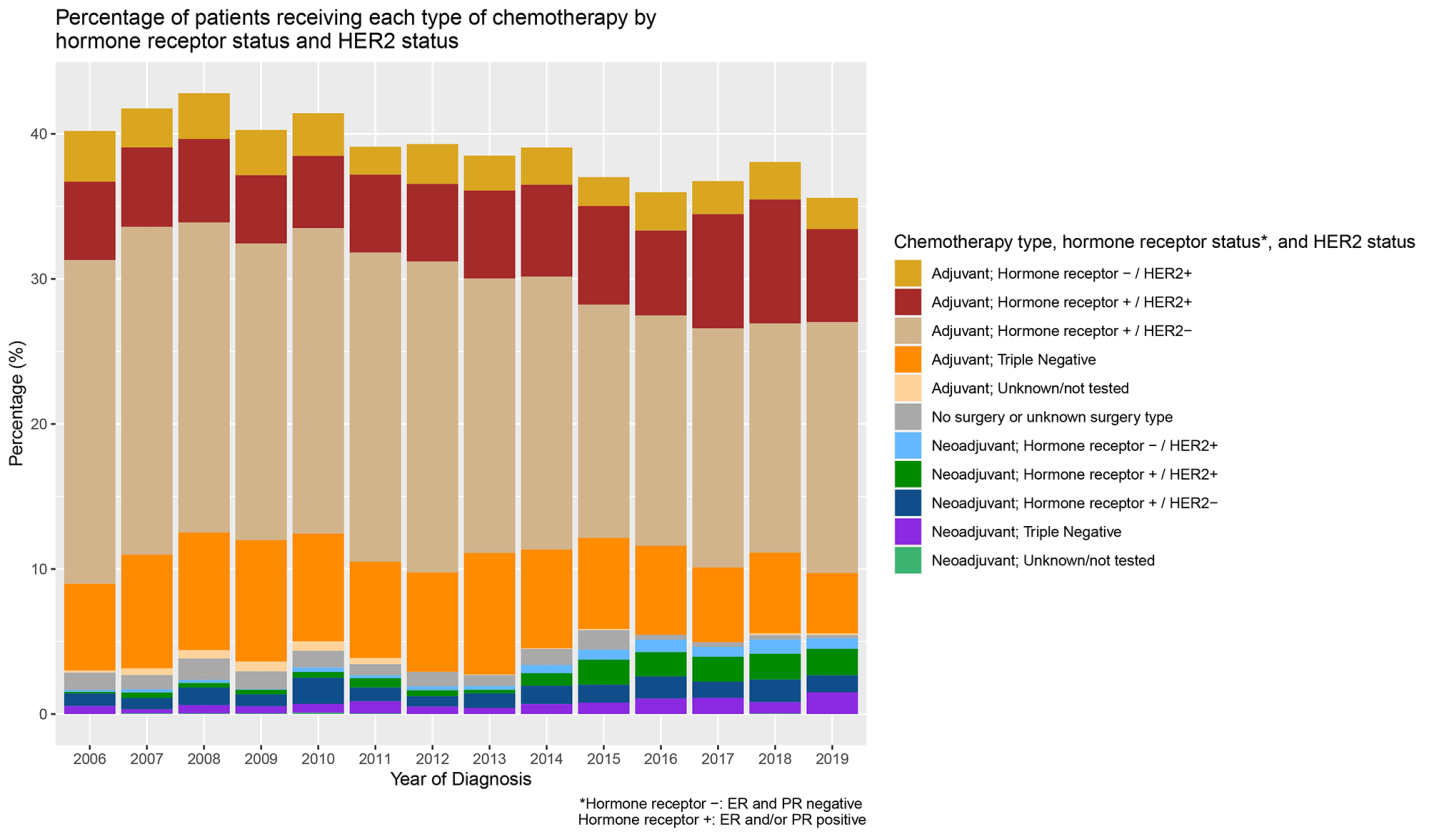
Figure shows among women receiving chemotherapy, how use of adjuvant and neoadjuvant chemotherapy has changed over time, relative to hormone receptor status and HER2 status. Note that in this graph, negative hormone receptor status refers to ER (estrogen receptor)- and progesterone receptor (PR) - disease, while hormone receptor positive status reflects ER+ and/or PR+ disease. Figure also shows the group who received chemotherapy but for whom there's no indication of receipt of surgery or for whom surgery data are missing, and thus are grouped separately

Overall, 13,089 (38.9%) women received chemotherapy (Table 1). Of the women receiving chemotherapy, 54.8% had stage II disease (with 32.3% and 13.0% of those having stage I and IIIA disease, respectively). The majority of participants were white (74.6%), with Asians making up 16.5% of the cohort and Black and African Americans making up 7.3%; 12.9% of the cohort is Hispanic. There was a decline in receipt of chemotherapy over time (40.2% in 2006 vs. 35.6% in 2019)(p-trend < 0.001)(Figs. 1 and 2). Of the women who received chemotherapy, 8.5% were known to have received neoadjuvant treatment overall, increasing from 4.1% in 2006 to 14.7% in 2019 (p-trend < 0.001) (Fig. 1), with some variation in the patterns over time by hormone receptor status/HER2 status (Fig. 2). Furthermore, of those who received chemotherapy, 27.1% received trastuzumab/pertuzumab, with receipt increasing over time (p-trend < 0.001): in 2006, 20.8% received trastuzumab and/or pertuzumab, increasing to 30.9% in 2019.

**Table 1:** Population characteristics, by receipt of chemotherapy

			Among those receiving chemotherapy	
	Overall	Participants who received chemotherapy (n, %)	Received trastuzumab and/or pertuzumab (n, %)	Did not receive trastuzumab and/or pertuzumab (n, %)
***TOTAL***	*33,670*	*13,089 (39%)*	*3,548 (27%)*	*9,541 (73%)*
Characteristic				
**Age at diagnosis (years)**				
18–39	1,509 (4.5%)	1,194 (9.1%)	365 (10.3%)	829 (8.7%)
40–49	5,283 (15.7%)	3,230 (24.7%)	802 (22.6%)	2,428 (25.4%)
50–64	13,656 (40.6%)	6,064 (46.3%)	1,619 (45.6%)	4,445 (46.6%)
65–79	10,568 (31.4%)	2,516 (19.2%)	715 (20.2%)	1,801 (18.9%)
80+	2,654 (7.9%)	85 (0.6%)	47 (1.3%)	38 (0.4%)
**Race**				
White	25,130 (74.6%)	9,313 (71.2%)	2,406 (67.8%)	6,907 (72.4%)
Black or African American	2,459 (7.3%)	1,106 (8.4%)	261 (7.4%)	845 (8.9%)
Asian	5,544 (16.5%)	2,430 (18.6%)	819 (23.1%)	1,611 (16.9%)
American Indian or Alaskan Native	136 (0.4%)	50 (0.4%)	11 (0.3%)	39 (0.4%)
Native Hawaiian or Other Pacific Islander	240 (0.7%)	120 (0.9%)	31 (0.9%)	89 (0.9%)
More than once race	55 (0.2%)	21 (0.2%)	9 (0.3%)	12 (0.1%)
Unknown/missing	106 (0.3%)	49 (0.4%)	11 (0.3%)	38 (0.4%)
**Ethnicity**				
Not Hispanic	29,341 (87.1%)	11,059 (84.5%)	3,037 (85.6%)	8,022 (84.1%)
Hispanic	4,328 (12.9%)	2,030 (15.5%)	511 (14.4%)	1,519 (15.9%)
**Year of diagnosis**				
2006–2010	11,056 (32.8%)	4,566 (34.9%)	974 (27.5%)	3,592 (37.6%)
2011–2015	12,482 (37.1%)	4,816 (36.8%)	1,329 (37.5%)	3,487 (36.5%)
2016–2019	10,132 (30.1%)	3,707 (28.3%)	1,245 (35.1%)	2,462 (25.8%)
**AJCC Stage** ^**a**^				
Stage I	20,199 (60.0%)	4,223 (32.3%)	1,445 (40.7%)	2,778 (29.1%)
Stage II	11,456 (34.0%)	7,170 (54.8%)	1,733 (48.8%)	5,437 (57.0%)
Stage IIIA	2,015 (6.0%)	1,696 (13.0%)	370 (10.4%)	1,326 (13.9%)
**Tumor subtype**				
Triple negative	3,523 (10.5%)	2,555 (19.5%)	25 (0.7%)	2,530 (26.5%)
Hormone receptor +/HER2 -	24,994 (74.2%)	6,955 (53.1%)	123 (3.5%)	6,832 (71.6%)
Hormone receptor +/HER2 +	3,166 (9.4%)	2,424 (18.5%)	2,344 (66.1%)	80 (0.8%)
Hormone receptor -/HER2 +	1,388 (4.1%)	1,067 (8.2%)	1,039 (29.3%)	28 (0.3%)
Unknown/not tested	599 (1.8%)	88 (0.7%)	17 (0.5%)	71 (0.7%)

*Abbreviations*

^**a**^AJCC: American Joint Committee on Cancer

^**b**^HER2: Human epidermal growth factor receptor 2

*Note* Hormone receptor + defined as ER + and/or PR+

**Fig. 3:**
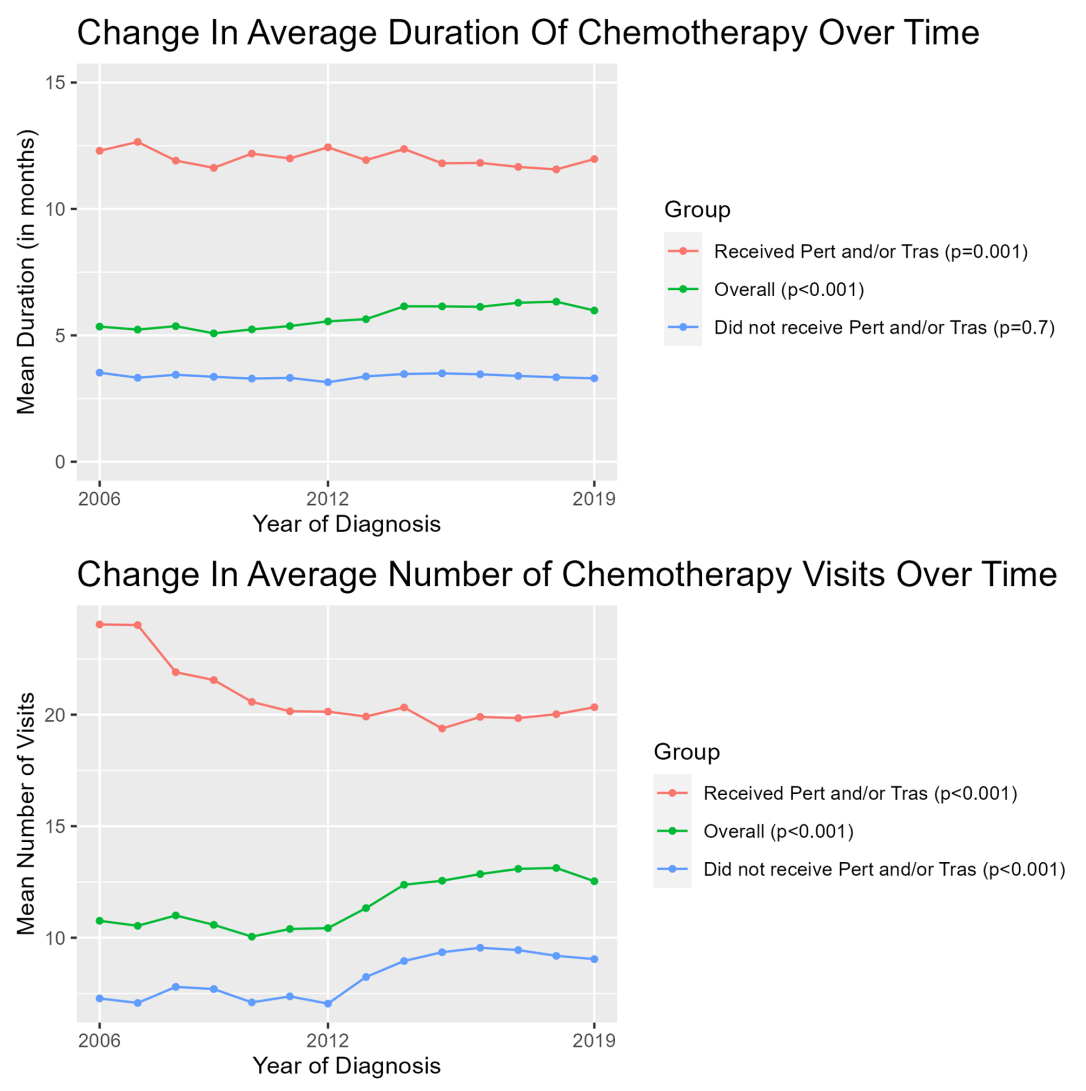
Among those receiving chemotherapy infusions, there has been an increase in duration of chemotherapy over time and an increase in the average number of chemotherapy visits over time. Among those receiving trastuzumab/pertuzumab, average duration of chemotherapy and average number of visits has declined. For those not receiving trastuzumab/pertuzumab, average number of chemotherapy visits has increased

The average duration of chemotherapy was 5.3 months in 2006, increasing to 6.0 months in 2019 (p-trend < 0.001) for all patients receiving chemotherapy. The average number of chemotherapy infusion visits (inclusive of cytotoxic drugs and/or anti-HER2 therapies) increased from 10.8 visits in 2006 to 12.5 in 2019 (p-trend < 0.001)(Fig. 3). For those receiving trastuzumab and/or pertuzumab, there was a very slight decrease in the treatment duration (12.3 to 12.0 months(p-trend = 0.001) and a decrease in average number of infusion visits (24.0 to 20.3 months)(p-trend < 0.001)(Fig. 3). Conversely, for those not receiving trastuzumab and/or pertuzumab, there was an increase average number of visits from 7.3 to 9.0 (p-trend < 0.001). There was no significant trend observed in treatment duration for those not receiving trastuzumab and/or pertuzumab.

## Discussion

The prevalence of chemotherapy use in this cohort has decreased over time, with a marked increase in neoadjuvant treatment, consistent with other studies conducted in US populations. (13–14) Previous studies have reported that the use of 21-gene recurrence scores that inform decisions to forgo chemotherapy in lower risk patients may be driving decreases in chemotherapy use in breast cancer patients. (15–16) The average time spent undergoing chemotherapy and the average number of visits has increased. While one may assume this is due to the increasing use of anti-HER2 therapies (which have a substantially longer treatment duration than other drugs used to treat EBC), it should be noted that trastuzumab and/or pertuzumab-containing regimens have decreased in length and administration visits over time, while the number of visits has increased among those receiving cytotoxic drugs. The decrease in treatment duration for those receiving anti-HER2 therapies may reflect more treatment discontinuation secondary to toxicity or patient preference, as well as changing perceptions regarding the ideal duration of anti-HER2 therapies.

The overall increases in infusion visits and the average duration of chemotherapy represent an increasing time commitment for patients and providers, as well as a greater burden on the healthcare system for treatment delivery. These trends may also reflect improvements in symptom management that facilitate longer duration of chemotherapy, or administrative challenges in chemotherapy delivery, resulting in longer gaps between cycles. Further research is needed to better understand the drivers behind these trends.

This study provides an overview of broad chemotherapy trends in a sizeable cohort. It has several strengths, most notably a large sample size over a longitudinal 14-year period in a diverse cohort of breast cancer patients. Patients treated in integrated healthcare delivery settings have been shown to be representative of such underlying populations; therefore results are likely to be more generalizable and inform broader health services research [15]. Given this setting, results are not influenced by changes in insurance coverage. There are several limitations to consider; although this study considers chemotherapy administration visits, it does not incorporate data on laboratory services or other health services utilization which could result from toxicities or monitoring. We were unable to comment on 21-gene recurrence score uptake throughout the study period, which may have driven the decline in chemotherapy over time. Our results reflect real-world data, which has some limitations in use. For example, we identified a very small number of individuals in this large cohort classified as HER2-,who received trastuzumab and/or pertuzumab. HER2 status is largely obtained from cancer registries and extracted from diagnostic biopsy specimens. We abstracted medical chart data to address/correct any misclassified data, and thus any discordance that remains, even minimally, may reflect the complexity of obtaining and classifying HER2 status for research (e.g., HER2 may be reclassified with subsequent surgical assessment, or may be classified by the primary tumor in the setting of bilateral disease). It may also reflect research around HER2 expression in breast cancer stem cells and subsequent treatment of HER2- patients with anti-HER2 therapy [17], or the heterogeneity expected when examining a large treatment dataset.

In summary, the trends reported provide a systems-level overview of changing patterns of chemotherapy over time, showing a reduction in the prevalence of chemotherapy and increased chemotherapy visits. These data are useful to show that the treatment burden of routine chemotherapy care is changing over time, which has implications for cost, and resource allocation and administrative planning for health systems. This may inform future research to understand treatment uptake and changing patterns of care including regimen selection and how regimens are administered/received, and highlights the need to understand how these factors impact patient outcomes.

## Data Availability

Data contain potentially identifiable information such as dates of events that cannot be shared openly without appropriate human subjects approval and data use agreements, but are available upon reasonable request from the corresponding author.

## References

[1] Hortobagyi GN. Developments in chemotherapy of breast cancer. Cancer: Interdisciplinary International Journal of the American Cancer Society. 2000 Jun 15;88(S12):3073–9. 10.1002/1097-0142(20000615)88:12+<3073::aid-cncr26>3.3.co;2-i 10898354

[2] Guidelines Detail, Accessed NCCN. March 15, 2023. https://www.nccn.org/guidelines/guidelines-detail.

[3] Piccart-Gebhart MJ, Procter M, Leyland-Jones B (2005). Trastuzumab after Adjuvant Chemotherapy in HER2-Positive breast Cancer. N Engl J Med.

[4] Slamon D, Eiermann W, Robert N (2011). Adjuvant trastuzumab in HER2-Positive breast Cancer. N Engl J Med.

[5] Dieci MV, Vernaci G, Guarneri V (2019). Escalation and de-escalation in HER2 positive early breast cancer. Curr Opin Oncol.

[6] Sacchini V, Norton L (2022). Escalating de-escalation in breast cancer treatment. Breast Cancer Res Treat.

[7] Pittman NM, Hopman WM, Mates M (2015). Emergency room visits and Hospital Admission Rates after curative chemotherapy for breast Cancer. JOP.

[8] Majka ES, Trueger NS (2023). Emergency Department visits among patients with Cancer in the US. JAMA Netw Open.

[9] Numico G, Cristofano A, Mozzicafreddo A (2015). Hospital Admission of Cancer patients: avoidable practice or necessary care?. PLoS ONE.

[10] Whitney RL, Bell JF, Tancredi DJ (2019). Unplanned hospitalization among individuals with Cancer in the Year after diagnosis. JOP.

[11] Ross TR, Ng D, Brown JS, et al. EGEMS (Wash DC). 2014;2(1):1049. 10.13063/2327-9214.1049. The HMO Research Network Virtual Data Warehouse: A Public Data Model to Support Collaboration. 10.13063/2327-9214.1049 PMC4371424 25848584

[12] Hornbrook MC, Hart G, Ellis JL (2005). Building a virtual Cancer Research Organization. JNCI Monogr.

[13] Mougalian SS, Soulos PR, Killelea BK (2015). Use of neoadjuvant chemotherapy for patients with stage I to III breast cancer in the United States: timing of chemotherapy in breast Cancer. Cancer.

[14] Killelea BK, Yang VQ, Mougalian S (2015). Neoadjuvant chemotherapy for breast Cancer increases the rate of breast conservation: results from the National Cancer Database. J Am Coll Surg.

[15] Tesch ME, Speers C, Diocee RM (2022). Impact of TAILORx on chemotherapy prescribing and 21-gene recurrence score–guided treatment costs in a population-based cohort of patients with breast cancer. Cancer.

[16] Dinan MA, Mi X, Reed SD, Lyman GH, Curtis LH (2015). Association between Use of the 21-Gene recurrence score assay and receipt of Chemotherapy among Medicare beneficiaries with early-stage breast Cancer, 2005–2009. JAMA Oncol.

[17] Gomez SL, Shariff-Marco S, Von Behren J (2015). Representativeness of breast cancer cases in an integrated health care delivery system. BMC Cancer.

